# A Toolbox for Tuberculosis (TB) Diagnosis: An Indian Multi-Centric Study (2006-2008); Evaluation of Serological Assays Based on PGL-Tb1 and ESAT-6/CFP10 Antigens for TB Diagnosis

**DOI:** 10.1371/journal.pone.0096367

**Published:** 2014-05-05

**Authors:** Philippe H. Lagrange, Satheesh K. Thangaraj, Rajeshwar Dayal, Alaka Deshpande, Nirmal K. Ganguly, Enrico Girardi, Beenu Joshi, Kiran Katoch, Vishwa M. Katoch, Manoj Kumar, Vemu Lakshmi, Marc Leportier, Christophe Longuet, Subbalaxmi V. S. Malladi, Deepali Mukerjee, Deepthi Nair, Alamelu Raja, Balambal Raman, Camilla Rodrigues, Pratibha Sharma, Amit Singh, Sarman Singh, Archana Sodha, Basirudeen Syed Ahamed Kabeer, Guy Vernet, Delia Goletti

**Affiliations:** 1 Service de Microbiologie, Hôpital Saint Louis, Paris, France; 2 BioMérieux, Marcy-l'Etoile, France and New Delhi, India; 3 SN Medical College, Agra, India; 4 Sir J.J. Group of Govt Hosp. & Grant Medical College, Mumbai, India; 5 Indian Council of Medical Research, New Delhi, India; 6 Department of Epidemiology and Preclinical Research, L. Spallanzani National Institute for Infectious Diseases, Rome, Italy; 7 National JALMA Institute of Leprosy & Other Mycrobacterial Diseases, Agra, India; 8 All India Institute of Medical Sciences, New Delhi, India; 9 Nizam's Institute of Medical Sciences, Hyderabad, India; 10 Fondation Mérieux, Lyon, France; 11 Safdarjung Hospital, New Delhi, India; 12 National Institute for Research in Tuberculosis (formerly Tuberculosis Research Center), Chetput, Chennai, India; 13 Microbiology Section, P D Hinduja Hospital & Medical Research Centre, Veer Savarkar Marg Mahim, Mumbai, India; Hopital Raymond Poincare - Universite Versailles St. Quentin, France

## Abstract

**Background:**

The aim of this multi-centric prospective study in India was to assess the accuracy of a serological test as an additional tool for diagnosing active tuberculosis (ATB). In particular, an assay based on ELISA using a phenolic glycolipid (PGL-Tb1) or a fusion protein (ESAT-6/CFP10) was compared to the tuberculin skin test (TST) and the microbiological results according to HIV status.

**Methods:**

Individuals with and without ATB and HIV infection were enrolled. Serology and TST results were analyzed *per se* and in combination with the microbiological data.

**Results:**

Among the 778 ATB patients, 102 were HIV-infected, 316 HIV-uninfected and 360 had an HIV-unknown status. Of the 945 non-ATB subjects, 559 were at low risk (community adults) and 386 at high risk of *M. tuberculosis* exposure. Among those with ATB, the sensitivity of ELISA-PGL-Tb1 for ATB was higher than that of ELISA-ESAT-6/CFP10, both in HIV-infected (72.3% versus 63.7%, p = 0.29) and HIV-uninfected/HIV-unknown groups (40.5% versus 28.6%; p<0.0001), whereas the specificity was around 91% for both tests. Sensitivity for ATB increased when the results of the two ELISA were combined, reaching 75.5% in the HIV-infected and 50.9% in the group of HIV-uninfected/HIV-unknown ATB, with a significant decrease of the global specificity (83.9%). Analyzing the ELISA results with the microbiological results, we observed that the sensitivity of both serology tests was independent of the ATB patients' smear microscopy (SM) status and grade. Combining the results of SM with both ELISA, the detection of ATB patients significantly increased (p<0.0001), particularly in those with extrapulmonary TB (up to 45.1%) or HIV infection (up to 83.3%). No significant association was observed between TST and serology results.

**Conclusions:**

In this prospective multi-centric study, the combination of two rapid tests, such as SM and serology, might be useful in detecting ATB, especially in HIV-infected patients.

## Introduction

Diagnosing active tuberculosis (ATB) accurately and rapidly is a key challenge for eradicating the TB epidemic [1], [2]. Sputum smear microscopy (SM), currently the only diagnostic test in most TB-endemic areas, has several limitations; in particular, the sensitivity compared with culture is variable [2]–[4], multiple patient visits are required [5]–[7], considerable technical training is necessary and the procedure is labor-intensive [1], [8].

Antibody detection tests (serological tests), used for the diagnosis of several infectious diseases, may potentially improve TB diagnosis. These tests measure the presence of specific antibodies (mostly IgG) directed against immunodominant antigens of the investigated pathogen. Compared to SM, antibody detection methods may enable rapid TB diagnosis, as these tests have the advantages of being quick (results can be available within hours) and technologically easy, requiring minimal training. In addition, these tests could be adapted to point-of-care formats that can be implemented at lower levels of health services in low- and middle-income countries [8]–[11].

The serology tests used for diagnosing TB have a long record in the TB literature, but have never been well-developed, due to their low diagnostic values with poor specificity and sensitivity [12]. Since the 1990s, newer approaches have been chosen using enzyme-linked immunosorbent assays (ELISA) and highly purified antigens or recombinant proteins [9], [13]. Improvement of their performances has been obtained by using several different antigens simultaneously [14], [15]. However, several assessments of these serological tests in Human Immunodeficiency Virus-infected (HIV) patients have been at best inconclusive [16], [17] mainly because HIV-TB co-infected patients have been shown to be poor serological responders to protein antigens [18]–[22].

Serological tests based on ELISA using a panel of non-protein antigens such as glycolipids specific for *M. tuberculosis* have been developed [23], [24] and evaluated [13], [25]–[31]. About 65–70% of HIV-TB co-infected patients had serum reactivity to at least one glycolipid antigen and maintained the diverse antibody repertoire previously observed in HIV-uninfected TB patients [32]. The reason why such antibody response is preserved, despite the decline of CD4 T-lymphocyte counts in HIV-TB patients, has been related to the novel CD1-restricted lipid antigen presentation pathway [33], [34]. This is in sharp contrast to the classical response to T-cell-dependent peptide antigens which are MHC restricted [35], [36].

A remaining key question is, to what extent can the disappointing performance of current serological tests be associated with a high background prevalence of latent TB infection (LTBI) in the evaluated settings? A partial answer has been reported showing that the fusion protein of ESAT-6/CFP10 has variable diagnostic values among subjects from low (Denmark), moderate (Brazil) and high TB incidence countries (Tanzania and Ethiopia) [37]. Furthermore, it has been reported that the levels of antibodies against several protein antigens, including the ESAT-6/CFP10, might increase before the occurrence of clinical symptoms and microbiological confirmation of ATB in HIV-TB co-infected patients [38]; similar results have also been observed using glycolipid antigens such as PGL-Tb1 [39]. A specific remark also concerns the recent findings that an additional cell-surface glycolipid, the lipoarabinomannan (LAM), can be detected in the concentrated urines of ATB patients with advanced HIV disease [40]. In these patients with low CD4 cell counts, the sensitivity of this LAM-based assay was significantly higher than the SM (around 25%).

Although *in vitro* amplification of mycobacterial target DNA by PCR-based methods can provide rapid results, until recently the technology was not fully standardized. It is now suitable for routine clinical practice for diagnosing PTB [41]. However, a high proportion of negative results to the GeneXpert Technology were observed in SM/culture-negative extrapulmonary TB [42]. SM-negative PTB are frequently found in HIV-infected patients who require invasive procedures to confirm a diagnosis [1].

In a recent meta-analysis, inconsistent estimates of sensitivity and specificity of serology tests for ATB diagnosis have been described [43]–[45]. It was reported that none of the serology tests achieved sufficient sensitivity to replace SM, but the combination of serology tests based on selected antigens was shown to provide higher sensitivities than single antigens. The authors also recommended that further research be done to determine whether combinations of SM and antibody detection with multiple antigens could improve case findings in high prevalence countries.

A single rapid assay for TB diagnosis, such as the SM, PCR or serology, will not detect all ATB cases, thus we studied new algorithms in an attempt to increase the ATB cases detection rate. In an effort to find the best approach for TB diagnosis in a TB-endemic country, as India, we enrolled subjects with or without active TB and tried to identify the right combination of diagnostic tools for the toolbox. The first part of our multi-centric prospective study was to assess the diagnostic value of several microbiological tools [46], whereas the aim of the second part was to assess the performance of the QFT-GIT and TST, in addition to microbiological results, as contributors for diagnosing ATB according to HIV status [47]. The aim of this third part was to assess the performance of a serological test based on ELISA using a phenolic glycolipid or recombinant fusion protein, in addition to microbiological and TST results, as contributors for detecting ATB, stratifying by HIV status.

## Materials and Methods

### Study populations

Patients with ATB and individuals without ATB were prospectively enrolled from January 2006 to July 2008 at different medical centers as described [46]. Written informed consent was obtained from all subjects before enrollment. The study was approved by the local ethical committees: the Institutional Ethical Committee of the Tuberculosis Research Centre in Chennai (TRC-IEC No: 2006005), the Institutional Review Board at Hinduja Hospital, Mumbai (No: 316-05-CR), the Institutional Ethical Committee of AIIMS, New Delhi (AIIMS-IEC No: A-35:05/10/2005), the Institutional Ethical Committee of the Safdarjung Hospital, New Delhi (No Sur./1/2007), the NIMS Institutional Ethics Committee (No. EC/264 (A)/2005) and the Ethical Committee of National JALMA Institute for Leprosy and other Mycobacterial Diseases, Agra (minutes: 27/04/2006).

In brief, clinical symptoms and radiological findings of the patients were first assessed independently by each clinician taking part in the enrollment. Three sputum samples were collected and processed. The SM was stained using the hot Ziehl-Neelsen method and the semi-quantitative yield of acid-fast-bacilli (AFB) was recorded according to WHO recommendations [48]. The 3 sputum samples were cultured in solid and liquid medium and the presence of *M.tuberculosis* in the positive culture samples was further confirmed by molecular Gen-probe based PCR. The number of days to obtain a positive culture was also recorded [46].

Non-ATB individuals were also enrolled as controls: blood donors, healthy community adults (HCA), healthy family contacts (HFC), health care workers (HCW-laboratory staff and nurses), hospitalized non-ATB patients and cured TB patients. Because the different areas of recruitment are highly endemic for TB, all subjects (except the blood donors) were asked to give one to three sputum samples and were subjected to radiological examinations to rule out the suspicion of ATB. All enrolled healthy individuals were stratified by risk for TB exposure. Subjects at high TB risk were included only if they agreed to participate in a six month follow-up to exclude ATB occurrence.

Each individual's data were then recorded at each site using a standardized questionnaire involving 3 files (clinical/radiological evaluation, clinical/radiological follow-up and laboratory analysis) for both ATB patients and non-ATB individuals. An individual clinical suspicion of TB score (CSTB) was given for each patient; this was made by the clinician in charge of the patient at the time of inclusion in the study and before knowing any microbiological and immunological results. Included individuals were classified into 3 categories: very high, high, and low, as previously reported [46]. CSTB was used as a pre-test evaluation before enrollment. However, the study and the data analysis were performed using samples with a definite diagnosis of ‘‘ATB’’ or ‘‘non-ATB’’.

### Serological assays for HIV diagnosis

The presence of HIV infection was diagnosed or ruled out by two commercial ELISA in serum (Retroquic Comb Aids-RS, Span Diagnostics India; HIV TRI-DOT, J. Mitra & Co, India). The results were scored as positive when the serum was positive by both tests. If a serum was reactive in only one ELISA, HIV-Western Blot was performed as a confirmatory test to rule out a false ELISA result.

### Serology tests for TB diagnosis

The complete ELISA protocol standardization and buffer formulations were made by one of us (TS) in the R&D Immunoassay Laboratory (R&D lab), bioMérieux SA, France [49]. In brief, assay standardization was made using a unique specific wash buffer formulated for the PGL-Tb1 glycolipid and the ESAT-6/CFP10 fusion protein. All the ELISA plate coating and reagent preparations were made centrally in the R&D lab and then distributed to each microbiology laboratory associated with the enrollment sites. The tests were performed after specific training conducted by one of us (TS). Testing for each serum sample was performed in duplicate wells: ELISA for the separate detection of anti-PGL-Tb1 and anti-ESAT6-CFP10 IgG antibody (with a common newly formulated protocol). A positive control, negative control and calibrator were included for each ELISA plate to avoid inter plate variation. Any serum with discordant results higher than 10% between the 2 results from the duplicate was re-tested. The mean optical density (OD) from the 2 wells was recorded for each serum. We then calculated the median OD plus inter-quartile range (IQR) for each group of ATB patients and non-ATB individuals. No ELISA results were communicated to the physician in charge of the patients before the end of the study, and the laboratory technicians performing the serum ELISA were not informed of the medical status of the patient whose sample was tested.

### Antigens

PGLTb1: purification of PGLTb1 was performed using column chromatography as described earlier [50]. A new method of purification was designed to improve the purity of PGL-Tb1. This part of the work was done by one of us (TS) in the R&D Lab, and is described in his PhD thesis [49]. ESAT-6/CFP10: this antigen is a recombinant fusion protein and was provided by P. Andersen (Staten Serum Institute, Copenhagen. Denmark).

### Data collection

After collection, the data were subsequently transferred to EPIINFO files by one of the authors (TS). Each file included the patient's characteristics, risk of TB, clinical symptoms, whether TST was performed or not, chest X-ray findings, the effect of a 10 day antibiotic trial, the final clinical diagnosis, CSTB, the final therapeutic intervention with the therapy initiation date and the anti-TB drugs prescribed. The last part of the file consisted of the treatment outcome obtained during the 6 to 9 month follow-up of each individual with ATB and the absence of clinical symptoms in the non-ATB control groups. Patients with both pulmonary and extrapulmonary localizations (infiltrate and pleural effusion, for instance) were classified as pulmonary TB. The specific biological file consisted of the microbiological and the immunological results: HIV status (HIV-infected, HIV-uninfected or HIV-unknown), and in a subgroup of HIV-infected patients, the number of CD4 and CD8 cell counts/µL; OD values obtained by the ELISA using the PGL-Tb1 antigen and fusion protein ESAT-6/CFP10.

ATB was defined as ‘‘confirmed’’ either microbiologically (after identification of *M.tuberculosis* in LJ and/or in BacT/ALERT MP culture and molecular tests) or by histology. Conversely, patients were classified as having ‘‘clinical" ATB if the diagnosis was only based on clinical and radiological criteria (after excluding other diseases), including appropriate responses to anti-tuberculous therapy.

### Statistical data analysis

The Mann-Whitney test was used to compare the OD values to understand the significance of difference, while the Fisher exact test and Chi-Square test were used to evaluate the significance of difference between the numbers of positives of different serology tests [51], [52].The Pearson correlation was used to evaluate the correlation between the OD values of different ELISA and also to understand the impact of parameters like age, sex, SM grade and time to positivity (TTP) of the liquid culture upon the individual OD values. A Receiver Operative Characteristic (ROC) analysis was performed to measure the diagnostic efficiency of the ELISA to each antigen and also to find the best OD cut-off value [53]–[55]. Sensitivity, specificity, positive predictive value (PPV), negative predictive value (NPV) and the likelihood ratio (LR) were calculated as recommended [56]. The analysis was carried out with SPSS v 20 for Windows (SPSS Italia SRL, Bologna, Italy), GraphPad Prism 6.0 software and EPIINFO 3.2.2 (CDC, Atlanta, USA).

## Results

### Populations enrolled and characteristics

We enrolled 2,213 individuals in the 2-year period. We deleted 75 patient files because they were incomplete. Additionally, 272 children were evaluated separately [57], and 143 subjects belonging to a satellite study without any serology test were excluded (partial results already reported) [58]–[60].

**Figure 1:**
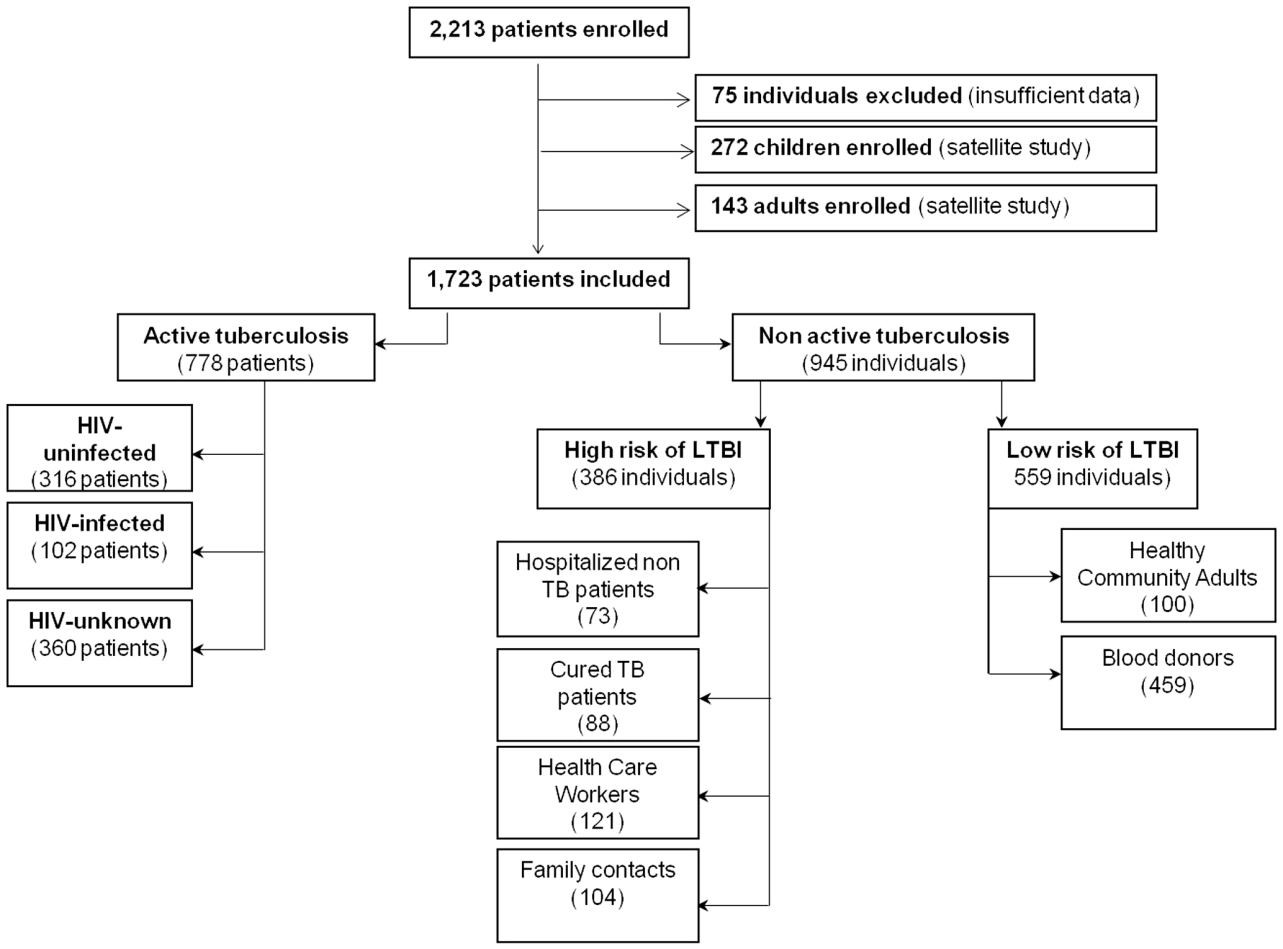
Flow chart of patients recruited to the multi-centric study stratified by active tuberculosis and non-active tuberculosis patient subgroups. **Footnotes**: HIV: Human Immunodeficiency Virus; LTBI: latent tuberculosis infection.

As shown in **Figure 1**, the remaining files correspond to 778 adult patients with ATB: 102 HIV-infected, 316 HIV-uninfected and 360 with an unknown HIV status. Based on the HIV status subgroup, the ATB patients were further stratified by TB localization (pulmonary and disease). The 945 individuals without ATB were further stratified by their relative risk of TB exposure: those at low risk of LTBI [459 healthy blood donors and 100 healthy community adults(HCA)] and those at high risk of LTBI [73 hospitalized non-active TB patients, 88 cured TB patients, 121 Health Care Workers (HCW), 104 Healthy Family contacts (HFC)].

### Serology results: quantitative ELISA results

**Figure 2:**
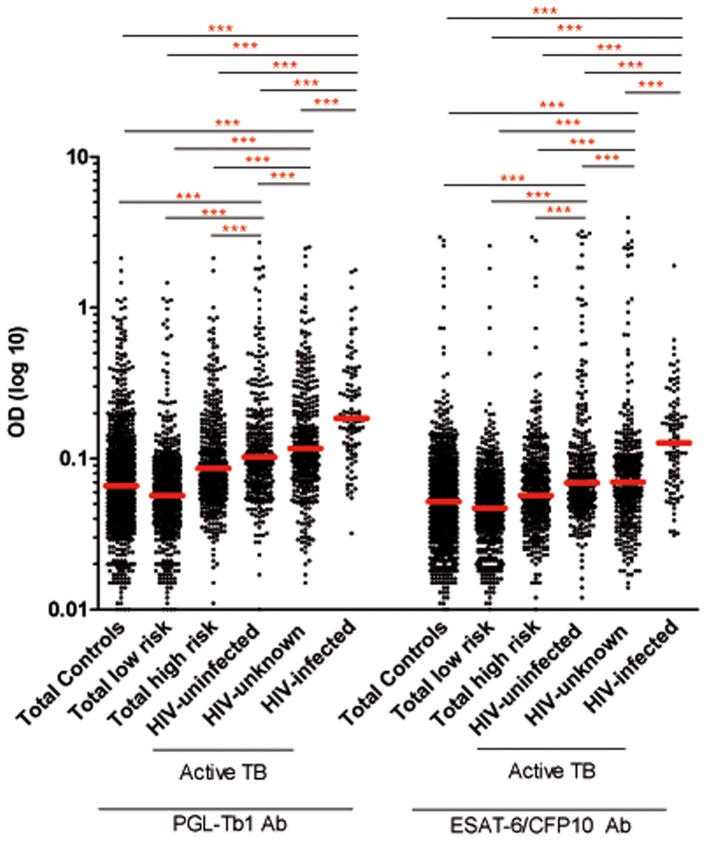
Individual IgG antibody levels against PGL-Tb1 and ESAT-6/CFP10 in subjects with or without active tuberculosis disease stratified by HIV status. Dot plot indicates the single antibody level per subject analyzed; median is indicated by the red line. ***: p<0.0001. **Footnotes**: HIV: Human Immunodeficiency Virus; LTBI: latent tuberculosis infection; OD: optical density.

**Figure 3:**
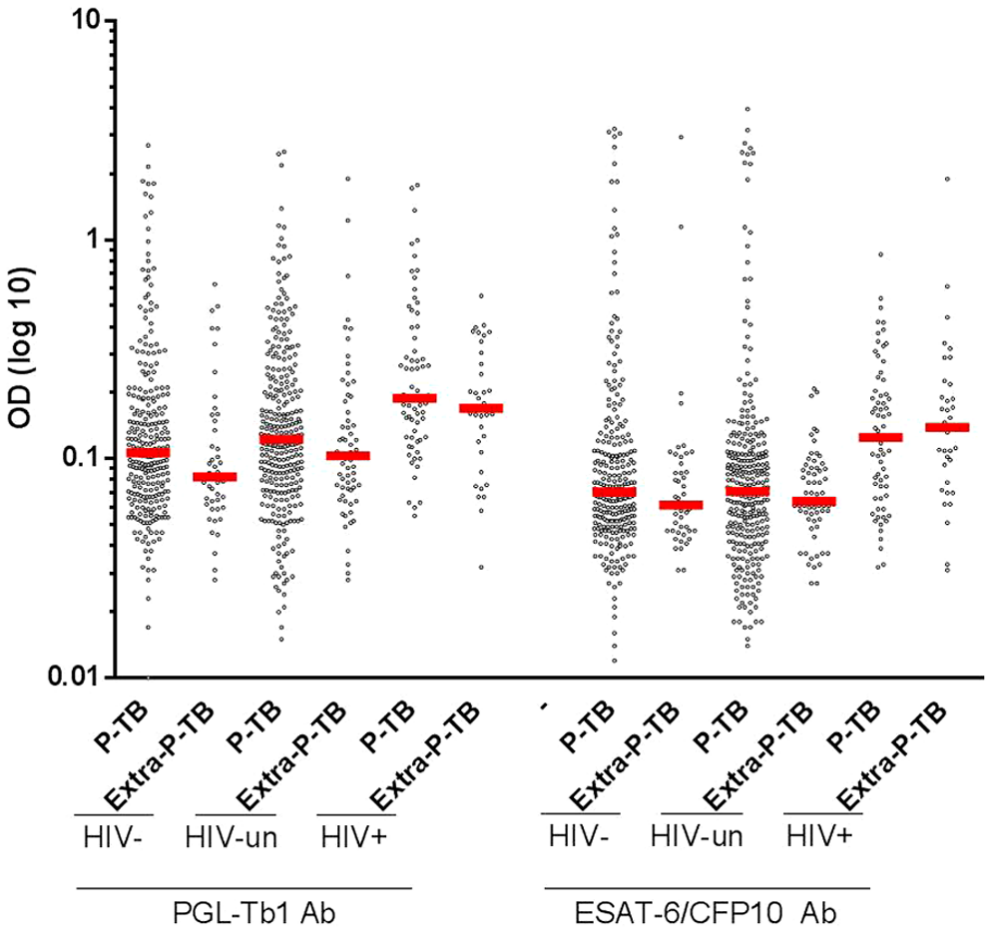
Individual IgG antibody levels against PGL-Tb1 and ESAT-6/CFP10 in subjects with active tuberculosis disease stratified by HIV status and disease localization (pulmonary and extrapulmonary). Dot plot indicates the single antibody level per subject analyzed; median is indicated by the red line. ***: p<0.0001. **Footnotes**: HIV: Human Immunodeficiency Virus; P-TB: pulmonary tuberculosis; Extra P-TB: extrapulmonary tuberculosis; OD: optical density.

The median anti-PGL-Tb1 and anti-ESAT6/CFP10 antibody levels were significantly higher in the different groups of ATB than in the low risk of LTBI group (*p*<0.0001) and the high risk LTBI group (p<0.0001) (**Table 1** **,** **Figure 2**), independent of the HIV status. The median anti-PGL-Tb1 and anti-ESAT6/CFP10 antibody levels were not significantly different between pulmonary and extrapulmonary ATB, independent of the HIV status, as shown in **Table 1** **,** **Figure 3**. When stratified by HIV status, the median anti-PGL-Tb1 and anti-ESAT6/CFP10 antibody levels were significantly higher among the 102 HIV-infected ATB patients than among the 316 HIV-uninfected (*p*<0.0001) or 360 HIV-unknown (*p*<0.0001) ATB patients. It is worthy to note that the median anti-PGL-Tb1 and anti-ESAT-6/CFP10 antibody levels between the HIV-unknown and the HIV-uninfected ATB patients were not statistically different (p = 0.0501 and p = 0.3173, respectively).

**Table 1:** Quantitative levels of anti-PGL-Tb1 and anti-ESAT-6/CFP10 antibodies (median) as measured by optical density in different groups of active TB patients according to their HIV status, disease localization and in individuals without active TB according to their risk of TB infection.

Active TB	HIV-infected			HIV-uninfected				HIV-unknown			
	N	Median OD (IQR)		N	Median OD (IQR)			N		Median OD (IQR)	
		PGL-Tb1	ESAT-6/CFP10		PGL-Tb1	ESAT-6/CFP10				PGL-Tb1	ESAT-6/CFP10
Pulmonary (P)	68	0.189	0.126	270	0.107	0.071		293		0.123	0.071
		(0.126–0.388)	(0.070–0.202)		(0.069–0.188)	(0.051–0.116)				(0.078–0.226)	(0.045–0.108)
									
Extrapulmonary (EP)	34	0.170	0.140	46	0.083	0.062		67		0.103	0.064
		(0.102–0.315)	0.077–0.221)		(0.062–0.152)	(0.047–0.097)				(0.073–0.163)	(0.053–0.090)
*p* P versus EP		*0.1895*	*0.6367*		*0.0549*	*0.1804*				*0.0886*	*0.4387*
All patients	102	0.185*	0.130*	316	0.103*	0.069*		360		0.117**	0.070**
		(0.124–0.347)	(0.074–0.208)		(0.067–0.172)	(0.049–0.108)				(0.076–0.214)	(0.046–0.105)
**No active TB**											
	N	Median OD (IQR)				**N**	Median OD (IQR)				
***Low risk of LTBI***		**PGL-Tb1**		**ESAT-6/CFP10**	***High risk of LTBI***		**PGL-Tb1**		**ESAT-6/CFP10**		
Blood donors	459	0.056		0.045	Hospitalized patients	73	0.077		0.060		
		(0.039–0.082)		(0.034–0.063)			(0.055–0.146)		(0.034–0.103)		
							
Healthy Community Adults	100	0.067		0.059	Health Care Workers	121	0.072		0.052		
		(0.046–0.100)		(0.043–0.080)			(0.052–0.106)		(0.039–0.081)		
							
					Healthy Family Contacts	104	0.089		0.053		
	-			-			(0.059–0.160)		(0.042–0.076)		
							
	-			*-*	Cured TB patients	88	0.112****		0.076****		
							(0.080–0.203)		(0.052–0.120)		
							
Total	559	0.057		0.047	Total	386	0.086***		0.057***		
		(0.040–0.085)		(0.035–0.067)			(0.057–0.149)		(0.041–0.087)		

**Footnotes**: TB: tuberculosis; HIV: human immunodeficiency virus; LTBI: latent tuberculosis infection; N; number of serum tested; OD: optical density; * HIV-infected versus HIV-uninfected, versus HIV-unknown: p<0.0001; ** HIV-uninfected versus HIV-unknown: p>0.05; *** Low risk versus high risk individuals*: p* <0.0001;****Cured TB patients versus other HR of LTBI: p<0.01; IQR (interquartile range).

**Figure 4:**
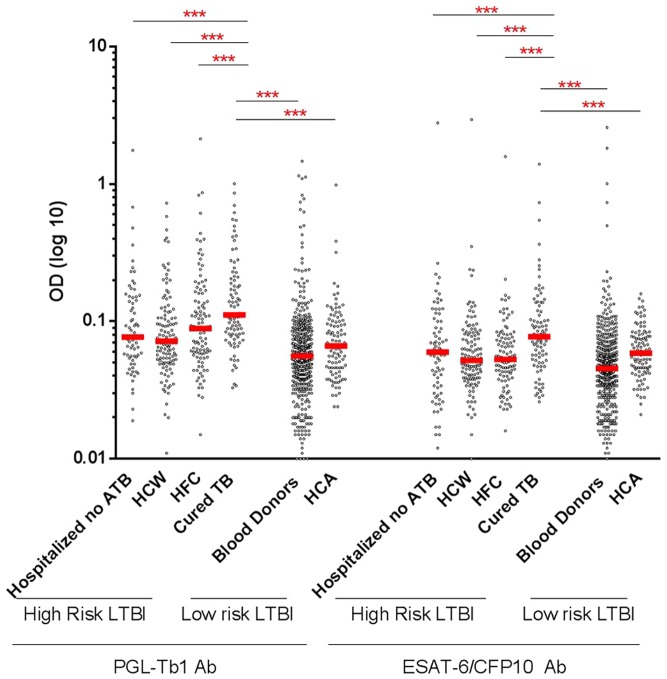
Individual IgG antibody levels against PGL-Tb1 and ESAT-6/CFP10 in subjects without active tuberculosis disease stratified by the risk of tuberculosis infection. Dot plot indicates the single antibody level per subject analyzed; median is indicated by the red line. ***: p<0.0001. **Footnotes**: LTBI: latent tuberculosis infection; Ab: antibody; ATB: active tuberculosis; HCW: health care workers; HFC: healthy family contacts; HCA: healthy community adults; OD: optical density.

Among the individuals at high risk of LTBI, the cured-TB patients had significantly higher anti-PGL-Tb1 and anti-ESAT-6/CFP10 antibody levels than the hospitalized non-TB patients (p = 0.0004 and p = 0.0088), HCW (p<0.0001 and p<0.0001) and HFC (p = 0.0059 and p<0.0001) (**Table 1** **,** **Figure 4**). Considering the groups at high or low risk as a whole, the median anti-PGL-Tb1 and anti-ESAT-6/CFP10 antibody levels were significantly higher in the 386 individuals at high risk of LTBI (**Table 1** **,** **Figure 2**) than among the 559 individuals at low risk of LTBI (p<0.0001). It is important to note that the anti-PGL-Tb1 and anti-ESAT-6/CFP10 antibody levels were significantly lower for all the subgroups at high risk of LTBI compared to the ATB patients with unknown HIV status (p<0.0001), except for the group of cured-TB patients and HIV-unknown ATB patients, where no significant difference was observed (p = 0.99 and 0.19, respectively).

According to the relative clinical, radiological and microbiological variability observed in populations recruited at the different sites [46], the antibody responses were further evaluated in ATB patients and non-ATB individuals stratified by the enrolment center. Given that TB localization, as shown above (**Table 1** **,** **Figure 3**), was not influencing the anti-PGL-Tb1 and anti-ESAT-6/CFP10 antibody levels, we then investigated the effect of the site of recruitment upon the antibody levels of the whole group of ATB patients stratified by HIV status: the HIV-infected group versus the group of subjects defined as HIV-uninfected and the HIV-unknown (grouped together), since these two groups had serology results with comparable antibody levels.

The results are shown in **Table 2**. Among the HIV-infected patients, the anti-PGL-Tb1 and anti-ESAT-6/CFP10 antibody levels were not influenced by the site of recruitment and no significant difference was observed among each considered group (**Table 3**), except for the patients recruited at AIIMS who showed significantly higher anti-PGL-Tb1 levels (p = 0.0073). Differently, among the HIV-uninfected/HIV-unknown subgroup, significantly higher anti-PGL-Tb1 antibody levels were observed among the ATB patients recruited at AIIMS and JALMA compared to the other groups (**Table 4**). Similarly, significantly higher anti-ESAT-6/CFP10 antibody levels were observed among the ATB patients recruited at Hinduja and AIIMS compared to the other groups (**Table 4**).

**Table 2:** Evaluation of the quantitative level of anti-PGL-Tb1 and anti-ESAT-6/CFP10 antibodies in patients with active TB stratified by HIV status and center of enrolment.

	HIV-infected			HIV-non-infected*		
		Median OD (IQR)			Median OD (IQR)	
Center	N	PGL-Tb1	ESAT-6/CFP10	N	PGL-Tb1	ESAT-6/CFP10
**Hinduja**	50	0.161 (0.119–0.260)	0.140 (0.084–0.203)	190	0.107 (0.075–0.160)	**0.085** (0.060–0.109)
**AIIMS**	29	**0.280** (0.169–0.458)	0.127 (0.066–0.247)	94	**0.124** (0.071–0.441)	**0.079** (0.049–0.146)
**Safdarjung**	1	0.845 (0.845–0.845)	0.310 (0.310–0.310)	119	**0.096** (0.055–0.182)	0.062 (0.035–0.091)
**JALMA**	0	NA	NA	99	**0.144** (0.091–0.255)	0.063 (0.038–0.101)
**NIMS**	4	0.301 (0.107–0.664)	0.133 (0.068–0.250)	49	0.118 (0.060–0.210)	0.054 (0.032–0.107)
**TRC**	18	0.173 (0.088–0.417)	0.094 (0.058–0.192)	125	0.103 (0.069–0.172)	0.067 (0.52–0.090)

**Footnotes**: TB: tuberculosis; HIV: human immunodeficiency virus; LTBI: latent tuberculosis infection; N; number of serum tested; OD: optical density; IQR (interquartile range).

*HIV non-infected corresponds to the true HIV-uninfected and HIV-unknown patients with active TB.

**Table 3:** Evaluation of the statistical difference of PGL-Tb1 and anti-ESAT-6/CFP10 antibodies in HIV-infected active TB patients stratified by the center of enrolment (as reported in Table 2).

	Centers							Centers					
Centers	Hinduja	AIIMS	Safdarjung	JALMA	NIMS	TRC	Centers	Hinduja	AIIMS	Safdarjung	JALMA	NIMS	TRC
	*p value for PGL-Tb1 antibodies*							*p value for ESAT6/CFP10 antibodies*					
**Hinduja**	NA	*0.0073*	NA	NA	*0.5385*	*0.9314*	Hinduja	NA	*0.5223*	NA	NA	*0.7351*	*0.1085*
**AIIMS**	-	NA	NA	NA	*0.8155*	*0.0810*	AIIMS	-	NA	NA	NA	*0.9316*	*0.3418*
**Safdarjung**	-	-	NA	NA	NA	NA	Safdarjung	-	-	NA	NA	NA	NA
**JALMA**	-	-	-	NA	NA	NA	JALMA	-	-	-	NA	NA	NA
**NIMS**	-	-	-	-	NA	*0.4971*	NIMS	-	-	-	-	NA	*0.5843*
**TRC**	-	-	-	-	-	NA	TRC	-	-	-	-	-	NA

**Footnotes**: p value was evaluated by comparing the quantitative levels of antibodies obtained in the different tests by the Mann-Whitney test.

**Table 4:** Evaluation of the statistical difference of PGL-Tb1 and anti-ESAT-6/CFP10 antibodies in HIV-uninfected and unknown active TB patients stratified by the center of enrolment (as reported in Table 2).

		Centers										Centers			
Antigen	Centers	Hinduja	AIIMS	Safdarjung	JALMA	NIMS	TRC	Antigen	Centers	Hinduja	AIIMS	Safdarjung	JALMA	NIMS	TRC
		*p value for PGL-Tb1 antibodies*								*p value for ESAT6/CFP10 antibodies*					
PGL-Tb1		Hinduja	AIIMS	Safdarjung	JALMA	NIMS	TRC	ESAT6/CFP10		Hinduja	AIIMS	Safdarjung	JALMA	NIMS	TRC
	**Hinduja**	NA	*0.0461*	*0.1282*	*0.0005*	*0.8466*	*0.7251*		**Hinduja**	NA	*0.8400*	*<0.0001*	*0.0002*	*0.0005*	*0.0006*
	**AIIMS**	-	NA	*0.0027*	*0.6982*	*0.1778*	*0.0639*		**AIIMS**	-	NA	*0.0003*	*0.0039*	*0.0061*	*0.0532*
	**Safdarjung**	-	-	NA	*0.0002*	*0.3139*	*0.2016*		**Safdarjung**	-	-	NA	*0.3706*	*0.7169*	*0.0752*
	**JALMA**	-	-	-	NA	*0.0699*	*0.0018*		**JALMA**	-	-	-	NA	*0.3546*	*0.1968*
	**NIMS**	-	-	-	-	NA	*0.8253*		**NIMS**	-	-	-	-	NA	*0.0604*
	**TRC**	-	-	-	-	-	NA		**TRC**	-	-	-	-	-	NA

**Footnotes**: p value was evaluated by comparing the quantitative levels of antibodies obtained in the different tests by the Mann-Whitney test.

The antibody level variability among the different groups of non-ATB individuals recruited at the different sites and stratified by LTBI risk is shown in **Table 5**.

**Table 5:** Evaluation of quantitative levels of anti-PGL-Tb1 and anti-ESAT-6/CFP10 antibodies (medians) in individuals with varying risk of latent tuberculosis infection stratified by the center of enrolment.

Antigen	Center		High risk controls								
			Hospitalized non-ATB Patients		Heath Care Workers			Healthy Family Contacts			Cured TB Patients
		N	OD median (IQR)	N	OD median (IQR)		N	OD median (IQR)		N	OD median (IQR)
**PGL-Tb1**	**Hinduja**	40	0.078 (0.057–0.157)	38	0.064 (0.052–0.093)		0	NA		0	NA
	**AIIMS**	2	0.104 (0.067–0.140)	34	0.064 (0.051–0.088)		0	NA		0	NA
	**Safdarjung**	1	0.171	0	NA		0	NA		0	NA
	**JALMA**	30	0.076 (0.049–0.106)	0	NA		48	0.083 (0.045–0.118)		0	NA
	**TRC**	0	NA	49	0.095* (0.053–0.173)		56	0.098* (0.063–0.187)		90	0.112 (0.080–0.203)
**ESAT-6/CFP10**	**Hinduja**	42	0.074 (0.055–0.124)	38	0.046 (0.038–0.070)		0	NA		29	NA
	**AIIMS**	2	1.419 (0.056–2.781)	34	0.054 (0.042–0.078)		0	NA		5	NA
	**Safdarjung**	1	0.070	0	NA		0	NA		0	NA
	**JALMA**	30	0.030* (0.025–0.061)	0	NA		48	0.044 (0.028–0.060)		0	NA
	**TRC**	0	NA	49	**0.055** (0.040–0.109)		56	0.064* (0.048–0.084)		90	0.076 (0.052–0.120)
**Low risk controls**											
			**Blood donors (BD)**			**Healthy Community Adults (HCA)**					
		**N**	**OD median (IQR)**	**N**		**OD median (IQR)**			***p*** ** value BD versus HCA**		***p value BD from Hinduja vs BD from other sites***
**PGL-Tb1**	**Hinduja**	199	0.065 (0.049–0.090)	100		0.067 (0.046–0.100)			*0.9000*		*NA*
	**AIIMS**	200	**0.051** (0.035–0.076)	0		NA			*NA*		*0.0003*
	**NIMS**	60	**0.039** (0.020–0.056)	0		NA			*NA*		*<0.0001*
**ESAT-6/CFP10**	**Hinduja**	199	0.057 (0.042–0.081)	100		0.059 (0.043–0.080)			*0.0737*		*NA*
	**AIIMS**	200	**0.042** (0.029–0.053)	0		NA			*NA*		*<0.0001*
	**NIMS**	69	**0.027** (0.018–0.041)	0		NA			*NA*		*<0.0001*

**Footnotes**: N =  number of serum tested; NA: not applicable because no individual has been recruited at that center;* p<0.01.

Among the low risk of LTBI group, the blood donors and the HCA enrolled at Hinduja showed similar anti-PGL-Tb1 and anti-ESAT-6/CFP10 antibody levels (p = 0.90 and p = 0.07, respectively), however these levels were significantly higher than those observed among the blood donors recruited at AIIMS (<0.0001) or at NIMS (*p*<0.0001).

Among the different subgroups of individuals at high risk for LTBI, there was an important variability associated with the antibody levels considered and the site of recruitment. For instance, the 30 hospitalized patients with lepromatous leprosy enrolled at JALMA showed significantly lower anti-ESAT-6/CFP10 antibody levels than the 47 hospitalized non-ATB patients enrolled at Hinduja (*p*<0.0001), but no significant difference was observed for the anti-PGL-Tb1 antibody levels (p = 0.25). Among the HCW group, the antibody levels against both antigens in those enrolled at Hinduja and at AIIMS were not statistically different (*p* = 0.92). In contrast, those enrolled at TRC presented higher anti-PGL-Tb1 and anti-ESAT-6/CFP10 antibody levels compared to the HCW enrolled at the 2 other centers, but the difference was significant only for the anti-PGL-Tb1 when compared with those enrolled at AIIMS (*p* = 0.0187). Among the HFC group, significantly higher anti-PGL-Tb1 and anti-ESAT-6/CFP10 antibody levels were found in those enrolled at TRC, compared to those enrolled at JALMA (*p = 0.0199*, *p*<0.0001, respectively).

### Qualitative ELISA results and diagnostic values for ATB. ROC curve analysis

Based on the significant difference found in the quantitative analysis of serological tests, we performed an ROC analysis to evaluate its potentials for TB diagnostics, stratifying by HIV status.

For this purpose, at the beginning we used ATB HIV-infected, HIV-uninfected or HIV-unknown individuals as the “diseased group” and the blood donors as the “control group”. In a further analysis of those with ATB, we combined the serological data from patients without HIV infection with those with an unknown HIV status.

**Figure 5:**
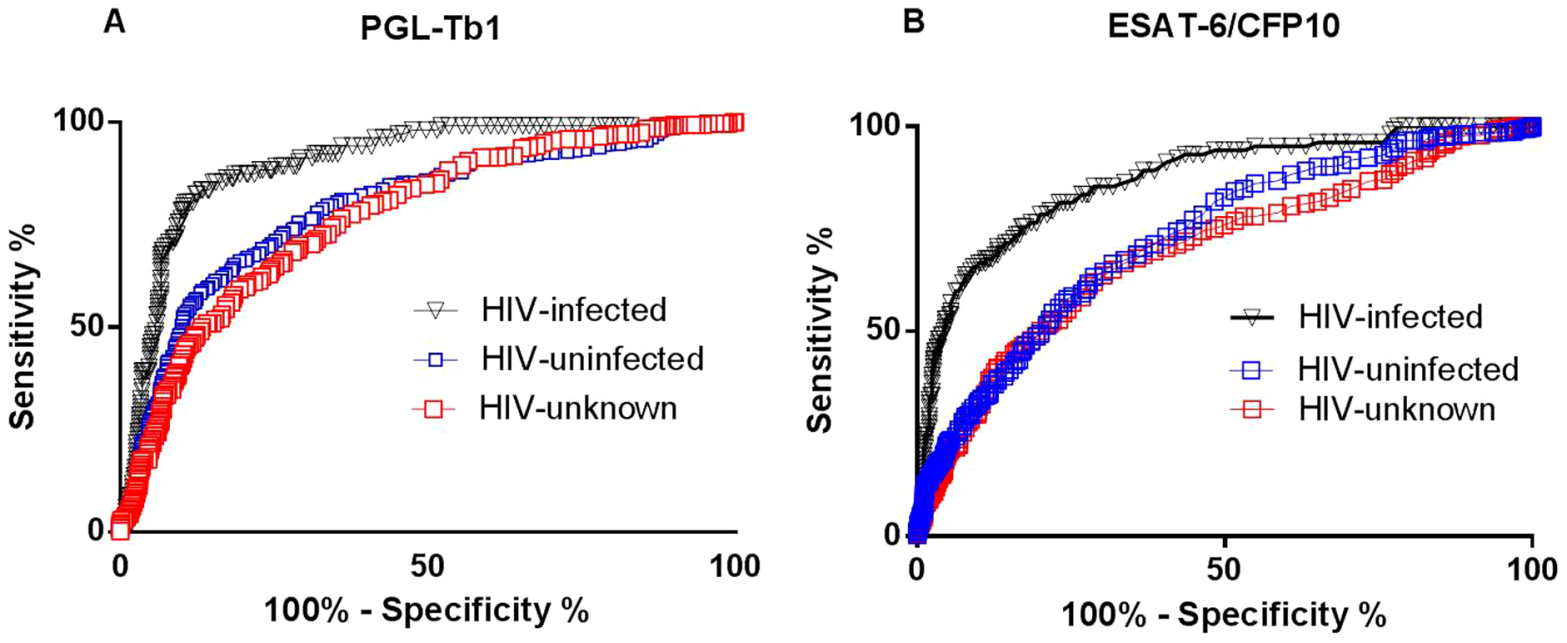
Receiving operative curves of a serology test for active tuberculosis in patients stratified by HIV status. Receiving operative curves calculated in HIV-uninfected (blue spot), HIV-unknown (red spot) or HIV-infected (black spot) patients with active TB and in healthy individuals with low risk of latent TB infection for antibodies: A. against PGL-Tb1 (left graph); B. ESAT-6/CFP10 (right graph) antigens. **Footnotes**: HIV: Human Immunodeficiency Virus.

Significant area under the curve (AUC) analysis results were obtained, independent of the antigen used and HIV status. It is important to note that the results obtained with anti-PGL-Tb1 antigen demonstrated a significantly higher discriminative ability in the group of HIV-infected ATB patients compared to the results obtained in the combined group (HIV-uninfected and HIV-unknown ATB patients), with higher AUC values (0.901 and 0.780). Similarly, for the results obtained with the ESAT-6/CFP10 antigen, a significantly higher discriminative ability was found in the HIV-infected ATB patients than in the combined group (HIV-uninfected and HIV-unknown ATB patients), with higher AUC values (0.869 and 0.713). Similar results were observed when HIV-uninfected and HIV-unknown groups were considered separately, **Table 6** **,** **Figure 5**).

**Table 6:** Evaluation of areas under the curve of the Receiver Operating Characteristic (ROC) of anti-PGL-Tb1 and anti-ESAT6/CFP10 antibodies of 102 HIV-infected, 316 HIV-uninfected, 360 HIV-unknown active TB patients and additional subjects without active TB (459 healthy subjects as in the blood donors).

Antigen	HIV-infected	HIV-uninfected	HIV-unknown	HIV-non-infected*	HIV-uninfected versus HIV-unknown	HIV-infected versus HIV-non-infected
	areas under the curve of the Receiver Operating Characteristic (95%CI)					
**PGL-Tb1**	0.901±0.015	0.770±0.017	0.790±0.016	0.780±0.014	*p = 0.40*	*p<0.0001*
	(0.870–0.931)	(0.736–0.803)	(0.758–0.822)	(0.753–0.808)	*p = 0.40*	*p<0.0001*
	*p*<0.0001	*p*<0.0001	*p*<0.0001	*p*<0.0001		
**ESAT-6/CFP10**	0.869±0.019	0.730±0.018	0.699±0.019	0.713±0.015	*p = 0.23*	*p<0.0001*
	(0.830–0.907)	(0.694–0.766)	(0.662–0.735)	(0.683–0.743)	*p = 0.23*	*p<0.0001*
	*p*<0.0001	*p*<0.0001	*p*<0.0001	*p*<0.0001		

**Footnotes**: TB: tuberculosis; HIV: human immunodeficiency virus; * HIV-non-infected corresponds to the true HIV-uninfected and HIV-unknown patients with active Tuberculosis.

### Diagnostic values: overall sensitivity for ATB diagnosis

By ROC analysis we found that the OD cut-off point of 0.130 for the PGL-Tb1 antigen (with an LR of 8.05 and 4.55 for the HIV-infected and HIV-non infected ATB patients, respectively) and an OD of 0.100 for the ESAT-6/CFP10 antigen (with an LR of 7.70 and 3.45 for the HIV-infected and HIV-non infected ATB patients, respectively) predicted ATB with the sensitivity and specificity indicated in **Table 7**.

**Table 7:** Sensitivity of the serology test using ELISA based on the PGL-Tb1 or the ESAT-6/CFP10 antigen and their cumulative results in the different groups of active TB enrolled patients stratified by HIV status and TB localization.

Antigen	HIV status	n/N	Pulmonary ATB	n/N	Extrapulmonary ATB%	Pulmonary versus extrapulmonary	n/N	All ATB%
			% (95% CI)		% (95% CI)			% (95% CI)
PGL-Tb1 *	Infected	50/68	**73.5** (61.4–83.5)***	23/34	**67.7** (49.5–82.6)***	*P = 0.6423*	73/102	**72.3** (62.4–80.7)***
	Uninfected	104/270	38.5 (32.7–44.6)	13/46	28.3 (16.0–43.5)	*P = 0.2472*	117/316	37.0 (31.7–42.6)
	Unknown	136/293	46.4 (40.6–52.3)	22/67	32.8 (21.9–45.4)	*P = 0.0556*	158/360	43.6 (38.4–48.9)
	Uninfected +unknown	240/563	42.6 (38.5–46.8)	35/113	31.0 (22.6–40.4)	*P = 0.0213*	275/676	40.5 (36.8–44.3)
	Total	290/631	**46.0** (42.0–49.9)	58/147	**39.5** (31.5–47.8)	*P = 0.1675*	348/778	**44.7** (41.2–48.3)
ESAT-6/CFP10**	Infected	42/68	**61.8** (49.2–73.3)***	23/34	**67.7** (49.5–82.6)***	*P = 0.6639*	65/102	**63.7** (53.6–78.0)***
	Uninfected	82/270	30.4 (24.9–36.2)	11/46	23.9 (12.6–38.8)	*P = 0.4841*	93/316	29.4 (24.5–34.8)
	Unknown	89/293	31.0 (25.8–36.7)	11/67	16.4 (8.5–27.5)	*P = 0.0162*	100/360	27.8 (23.2–32.7)
	Uninfected +unknown	171/563	30.6 (26.8–34.5)	22/113	19.5 (12.6–28.0)	*P = 0.0169*	193/676	28.6 (25.2–32.1)
	Total	213/631	**33.9** (30.2–37.8)	45/147	**30.6** (23.3–38.7)	*P = 0.4966*	258/778	**33.2** (29.9–36.6)
**Both antigens**	Infected	52/68	**76.5** (64.6985.9)***	25/34	**73.5** (55.6–87.1)***	*P = 0.8088*	77/102	**75.5** (66.0–83.5)***
	Uninfected	133/270	49.3 (43.2–55.4)	20/46	43.5 (28.9–58.9)	*P = 0.5248*	153/316	48.4 (42.8–54.1)
	Unknown	166/293	56.7 (50.8–62.4)	25/67	37.3 (25.8–50.0)	*P = 0.0045*	191/360	53.1 (47.8–58.3)
	Uninfected +unknown	299/563	53.1 (48.9–57.3)	45/113	39.8 (30.7–49.5)	*P = 0.0131*	344/676	50.9 (47.1–54.7)
	Total	351/631	**55.6** (51.7–59.6)	70/147	**47.6** (39.3–56.0)	*P = 0.0816*	421/778	**54.1** (50.5–57.7)

**Footnotes**: TB: tuberculosis; HIV: human immunodeficiency virus; n/N = number of positive tests/number of tested sera in each group; * Cut-off value for PGL-Tb1: OD = 0.130; ** Cut-off value for ESAT-6/CFP10: OD = 0.100; *** HIV-infected versus HIV-uninfected or HIV-unknown: p<0.0001;

The sensitivity of the ELISA varied according to the tested populations and the antigen used. Such variability indicates the heterogeneity of the serological response, as already described [17], [61].

Independent of HIV status, the sensitivity of the ELISA using the PGL-Tb1 was significantly higher than that obtained with the ESAT6/CFP10 antigen for the entire group of ATB individuals (*p*<0.0001) and for the pulmonary ATB individuals (*p*<0.0001), but the difference was not significant for the extrapulmonary ATB Individuals (*p* = 0.1422). Similarly, the sensitivity of the ELISA using the PGL-Tb1 was significantly higher than that obtained with the ESAT6/CFP10 antigen for the entire group of HIV-uninfected (p<0.0001) or HIV-unknown (p<0.0001), but was not significantly higher when compared with the entire group of HIV-infected ATB patients (p = 0.2948).

The cumulative sensitivity obtained with the combination of the positive scores obtained by the two serology tests was significantly higher than the sensitivity obtained with each single test for all the ATB patients (*p* = 0.0003 and *p*<0.0001), and pulmonary ATB patients (*p* = 0.0007 and *p* = <0.0001). The difference varied more for the extrapulmonary ATB patients (PGL-Tb1: *p* = 0.1956 and ESAT6/CFP10: *p* = 0.0040).

Using the combined ELISA positive results from each antigen, the cumulative results showed that as a group, the overall sensitivity was significantly higher in the HIV-infected (75.5%) than in the HIV-uninfected (48.4%) (*p*<0.0001) or HIV-unknown (53.1%) ATB patients (*p*<0.0001), and there was no significant difference between the HIV-uninfected and HIV-unknown ATB patients (*p* = 0.2477). Regarding TB localization, the sensitivity was not statistically different in pulmonary (76.5%) or extrapulmonary (73.5%) HIV-infected ATB patients (p = 0.8088). The sensitivity was lower in the HIV-uninfected patients with extrapulmonary TB compared to those with pulmonary TB (43.5% and 49.3%, respectively) or in HIV-unknown patients (37.3% and 56.7%, respectively). The difference was not significant for the former (*p* = 0.5248), but statistically significant for the latter (*p* = 0.0045). When the whole group of HIV-uninfected and HIV-unknown patients was considered, the sensitivity was significantly higher in those with pulmonary ATB than extrapulmonary ATB (53.1% and 39.8%, respectively; p = 0.0131).

### Overall specificity for ATB diagnosis

The specificity for ATB diagnosis of the ELISA was almost 90.0% and quite similar for the 2 antigens considered when evaluated either in the blood donors or in the HCA group, with no significant difference observed between the two ELISA and the population tested (**Table 8**). To note: when the serology results from the two low risk populations were combined, the overall specificity for the PGL-Tb1 antigen (90.9%) was not significantly different than that obtained with the ESAT-/CFP10 antigen (91.4%) (*p* = 0.8334).

**Table 8:** Specificity of the serology test based on the ELISA using the PGL-Tb1 or the ESAT-6/CFP10 antigens and their cumulative results in the different groups of individuals classified as low risk of TB infection.

Antigen	n/N	Blood donors	n/N	Healthy subjects	*p* value of blood donors versus Healthy subjects	n/N	Total
	Percentage (95% CI)						
PGL-Tb1*	418/459	**91.1** (88.0–93.4)	90/100	**90.0** (82.4–94.5)	*p = 0.7038*	508/559	**90.9** (88.2–93.1)
ESAT-6/CFP10**	421/459	**91.7** (88.8–94.0)	90/100	**90.0** (82.4–94.5)	*p = 0.5573*	511/559	**91.4** (88.7–93.5)
PGL-Tb1 versus ESAT-/CFP10 (*p* value)		*p = 0.8141*		*p = 1.000*			*p = 0.8334*
**Both antigens**	389/459	**84.8** (81.1–87.9)	80/100	**80.0** (70.9–86.8)	*p = 0.2336*	469/559	**83.9** (80.6–86.9)

**Footnotes**: n/N = number of negative tests/number of tested sera in each group; * Cut-off value for PGL-Tb1: OD = 0.130; ** Cut-off value for ESAT-6/CFP10: OD = 0.100.

When the cumulative serology-positive results were used, the respective specificity declined significantly from 91.1% and 91.7% to 84.8% among the blood donors (*p* = 0.0044 and *p* = 0.0014), and from 90.0% to 80.0% among the HCA group, but the difference was not statistically significant (*p = *0.0734). The overall specificity using the cumulative positive ELISA results of both groups and both antigens declined significantly from 90.9% and 91.4% to 83.9% (*p* = 0.0006 and *p* = 0.0002, respectively); these 3 values were used to determine the respective diagnostic values of each ELISA and their cumulative results.

### Performances of the two ELISA for the diagnosis of ATB

The diagnostic values (PPV and NPV and LR) of each serological test and their cumulative results have been calculated and the results are shown in **Table 9**. The NPV and the positive LR were significantly higher among the HIV-infected compared to the HIV-non-infected ATB patients using the PGL-Tb1, ESAT-/CFP10, or using the cumulative results. By contrast, the PPV and the negative LR were significantly lower among the HIV-infected than the HIV-non-infected ATB patients using the PGL-Tb1, ESAT-/CFP10, or using the 2 antigens.

**Table 9:** Accuracy of active tuberculosis diagnosis by the serology test based on the ELISA using the PGL-Tb1, ESAT-6/CFP10 or both antigens in ATB patients (pulmonary and extrapulmonary TB) stratified by HIV status.

PGL-Tb1*	HIV-infected	HIV-infected + HIV-unknown	Total
**Sensitivity (95% CI)**	72.3 (62.4–80.7)	40.5 (36.8–44.3)	44.7 (41.2–48.3)
**Specificity (95% CI)**	90.9 (88.2–93.1)	90.9 (88.2–93.1)	90.9 (88.2–93.1)
**PPV (95% CI)**	58.9 (49.7-67.6)	84.3 (79.9–88.1)	87.2 (83.5–90.3)
**NPV (95% CI)**	94.8 (92.5–96.5)	55.8 (52.5–59.1)	54.2 (50.9–57.4)
**LR+**	7.93	4.44	4.90
**LR-**	3.31	1.53	1.65

ESAT-6/CFP10**	HIV-infected	HIV-infected + HIV-unknown	Total
**Sensitivity (95% CI)**	63.7 (53.6–78.0)	28.6 (25.2–32.1)	33.2 (29.9–36.6)
**Specificity (95% CI)**	91.4(88.8–93.6)	91.4(88.8–93.6)	91.4(88.8–93.6)
**PPV (95% CI)**	57.5 (47.9–66.8)	80.1 (74.5–84.9)	84.3 (79.9–88.2)
**NPV (95% CI)**	93.3 (90.8–95.2)	51.4 (48.3–54.6)	49.6 (46.5–52.7)
**LR+**	7.42	3.33	3.86
**LR-**	2.52	1.07	1.37

Both antigens	HIV-infected	HIV-infected + HIV-unknown	Total
**Sensitivity (95% CI)**	75.5 (66.0–83.5)	50.9 (47.14–54.7)	54.1 (50.5–57.7)
**Specificity (95% CI)**	83.9 (80.6–86.9)	83.9 (80.6–86.9)	83.9 (80.6–86.9)
**PPV (95% CI)**	46.1 (38.4–54.0)	79.3 (75.1–83.0)	82.4 (78.8-85.6)
**NPV (95% CI)**	94.9 (92.6–96.7)	58.6 (55.1–62.0)	56.8 (53.3–60.2)
**LR+**	4.69	3.16	3.36
**LR-**	3.43	1.71	1.83

**Footnotes**: TB: tuberculosis; HIV: human immunodeficiency virus; n/N = number of positive tests/number of tested sera in each group; * Cut-off value for PGL-Tb1: OD = 0.130; ** Cut-off value for ESAT-6/CFP10: OD = 0.100; CI: confidence interval; PPV: positive predictive value; NPV: negative predictive value; LR+: positive likelihood ratio; LR-: negative likelihood ratio.

### ELISA diagnostic values compared to Smear Microscopy (SM) results

In general, the serological tests have been described as having a very low diagnostic value for detecting SM-negative pulmonary TB patients [43], [62], therefore the sensitivity of the serology using ELISA based on two single antigens and their cumulative results was further evaluated in the ATB patients stratified by the SM results, HIV status and TB localization (**Table 10**).

**Table 10:** Sensitivity of the serology test based on the ELISA using the PGL-Tb1 and ESAT-6/CFP10 antigens and their cumulative results in active TB patients stratified by Smear Microscopy (SM), TB localization and HIV status.

Antigen	HIV status	Pulmonary				*p value SM(−) versus SM(+)*	Extrapulmonary				*p valueSM(−) versus SM(+)*
		n/N	SM-negative	n/N	SM-positive		n/N	SM-negative	n/N	SM-positive	
			% (95% CI)		% (95% CI)			% (95% CI)		% (95% CI)	
PGLTb1*	Infected	31/40	77.5 (61.6–89.2)	19/28	67.9 (47.7–84.1)	*0.4130*	22/30	73.3 (54.1–87.7)	2/4	50.0 (9.6–93.2)	*0.5636*
	Uninfected	19/64	29.7 (18.9–42.4)	85/206	41.3 (34.5–48.3)	*0.1072*	11/37	29.0 (15.4–45.9)	2/9	22.2 (2.8–60.0)	*1.0000*
	Unknown	49/113	43.4 (34.1–53.0)	87/180	48.3 (40.855.9)	*0.4704*	19/55	34.6 (22.2–48.6)	3/12	25.0 (5.5–57.2)	*0.7371*
	Uninfected +unknown	68/177	38.4 (31.2–46.0)	172/386	44.6 (39.5–49.7)	*0.1989*	30/92	32.3 (22.9–42.8)	5/21	23.8 (8.2–47.2)	*0.6022*
	**Total**	**99/217**	**45.6 (38.9–52.5)**	**191/414**	**46.1 (41.3–51.1)**	***0.9331***	**52/122**	**42.3 (33.4–51.5)**	**7/25**	**28.0 (12.1–49.4)**	***0.2626***
ESAT6/CFP10**	Infected	28/40	70.0 (53.5–83.4)	14/28	50.9 (30.7–69.4)	*0.1294*	20/30	66.7 (47.2–82.7)	3/4	75.0 (19.4–99.4)	*1.0000*
	Uninfected	21/64	32.8 (21.6–45.7)	62/206	30.1 (23.9–36.9)	*0.7567*	9/37	24.3 (11.8–41.2)	2/9	22.2 (2.8–60.0)	*1.0000*
	Unknown	30/113	26.6 (18.7–35.7)	59/180	32.8 (26.0–40.2)	*0.2972*	9/55	16.4 (7.7–28.8)	2/12	16.7 (2.1–48.4)	*1.0000*
	Uninfected +unknown	51/177	28.8 (22.3–36.1)	121/386	31.4 (26.8–36.2)	*0.5563*	18/92	19.6 (12.0–29.2)	4/21	19.1 (5.5–41.9)	*1.0000*
	**Total**	**79/217**	**36.4 (30.0–43.2)**	**135/414**	**32.6 (28.1–37.4)**	***0.3761***	**38/122**	**31.2 (23.1–40.2)**	**7/25**	**28.0 (12.1–49.4)**	***0.8166***
**Both antigens**	Infected	33/40	82.5 (67.2–92.7)	19/28	67.9 (47.7–84.4)	*0.2451*	23/30	73.3 (54.1–87.7)	3/4	75.0 (19.4–99.4)	*1.0000*
	Uninfected	28/64	43.8 (31.4–56.7)	104/102	50.5 (43.5–57.5)	*0.3914*	16/37	43.2 (27.1–60.5)	4/9	44.4 (13.7–78.8)	*1.0000*
	Unknown	56/113	49.6 (40.0–59.1)	111/180	61.7 (54.1–68.8)	*0.0523*	22/55	40.0 (27.0–54.1)	3/12	25.0 (5.5–57.2)	*0.5120*
	Uninfected +unknown	84/177	47.5 (39.9–55.1)	215/386	55.7 (50.6–61.7)	*0.0699*	38/92	46.3 (35.3–57.7)	7/21	33.3 (14.6–57.0)	*0.3313*
	**Total**	**117/217**	**53.9 (47.0–60.7)**	**234/414**	**56.6 (51.6–61.4)**	***0.5554***	**61/122**	**50.0 (40.8–59.2)**	**10/25**	**40.0 (23.1–61.3**	***0.3884***

**Footnotes**: TB: tuberculosis; HIV: human immunodeficiency virus; n/N = number of positive tests/number of tested sera in each group; SM: smear microscopy; * Cut-off value for PGL-Tb1: OD = 0.130; ** Cut-off value for ESAT-6/CFP10: OD = 0.100; CI: confidence interval.

It is clearly shown that the serology using each antigen performed equally well in SM-negative compared to SM-positive ATB patients, independent of their HIV status and TB localization (**Table 10**). To note: among the HIV-infected in whom the proportion of SM-positive results was very low (pulmonary TB: 41.2% and extrapulmonary TB: 11.8%), the cumulative ELISA results were able to detect 82.5% of the pulmonary cases and 73.3% of the extrapulmonary TB cases.

The combinations of results from SM status and combined serology tests were shown to improve the ATB case findings significantly, but varied according to the HIV status and TB disease localization (**Table 11**). The overall TB detection rate reached nearly 80% among the total ATB population, and the increased capacity to detect ATB was higher in extrapulmonary (+41.1%) compared to pulmonary TB patients (+14.9%). The increasing capacity to detect ATB patients was even higher among the HIV-infected than among the HIV-uninfected or HIV-unknown ATB patients. The highest case finding was obtained among both the pulmonary (89.7%) and extrapulmonary HIV-infected ATB patients (79.4%). Similar results were also observed using the results obtained with single antigens (data not shown).

**Table 11:** Sensitivity of the SM test and combined sensitivity of the SM with the serology results based on the cumulative anti-PGL-Tb1 and anti-ESAT-6/CFP10 ELISA positive data among the SM-negative (serology positive) active TB patients stratified by disease localization and HIV status.

HIV status	test	n/N	Pulmonary TB	n/N	Extrapulmonary TB	n/N	All forms of TB
			% (95% CI)		% (95% CI)		% (95% CI)
**Infected**	SM (+)	28/68	41.2 (29.4–53.8)	4/34	11.8 (3.3–27.5)	32/102	31.4 (22.6–41.3)
	SM(+) + ELISA (+)	61/68	**89.7** (79.9–95.8)	27/34	**79.4** (62.1–91.3)	88/102	**86.3** (78.0–92.3)
	*SM+ versus SM+ELISA+*		*p <0.0001*		*p <0.0001*		*p <0.0001*
**Uninfected**	SM (+)	206/270	76.3 (70.6–81.2)	9/46	19.6 (9.4–33.9)	215/316	68.0 (62.6–73.2)
	SM(+) + ELISA (+)	234/270	**86.7** (82.2–90.5)	25/46	**54.4** (39.0–69.1)	259/316	**82.0** (77.3–86.0)
	*SM+ versus SM+ELISA+*		*p = 0.0027*		*p = 0.0010*		*p <0.0001*
**Unknown**	SM (+)	180/293	61.4 (55.6–67.0)	12/67	17.9 (9.6–29.2)	192/360	53.3 (48.0–58.6)
	SM(+) + ELISA (+)	236/293	**80.5** (75.5–84.9)	34/67	**50.8** (38.2–63.2)	270/360	**75.0** (70.2–79.4)
	*SM+ versus SM+ELISA+*		*p <0.0001*		*p = 0.0001*		*p <0.0001*
**Uninfected +Unknown**	SM (+)	386/563	68.6 (64.6–72.4)	21/113	18.6 (11.9–27.0)	407/676	60.2 (56.4–63.9)
	SM(+) + ELISA (+)	470/563	**83.5** (80.2–86.5)	59/113	**52.2** (42.6–61.7)	529/676	**78.3** (80.0–81.3)
	*SM+ versus SM+ELISA+*		*p <0.0001*		*p <0.0001*		*p <0.0001*
**Total**	SM (+)	414/631	65.6 (61.8–69.3)	25/147	17.0 (11.3–24.1)	439/778	56.4 (52.9–60.0)
	SM(+) + ELISA (+)	531/631	**84.2** (81.1–86.9)	86/147	**58.1** (49.7–66.2)	617/778	**79.3** (76.3–82.1)
	*SM+ versus SM+ELISA+*		*p <0.0001*		*p <0.0001*		*p <0.0001*

**Footnotes**: TB: tuberculosis; HIV: human immunodeficiency virus; n/N = number of positive tests/number of tested sera in each group; SM: smear microscopy; Cut-off value for PGL-Tb1: OD = 0.130; ESAT-6/CFP10: OD = 0.100.

The increasing capacity to detect ATB patients using the combination of the results from SM and serology in ATB patients appeared to be related to the independence of the antibody production relative to the TB localization as shown in **Table 1** **and** **Table 10**.

**Figure 6:**
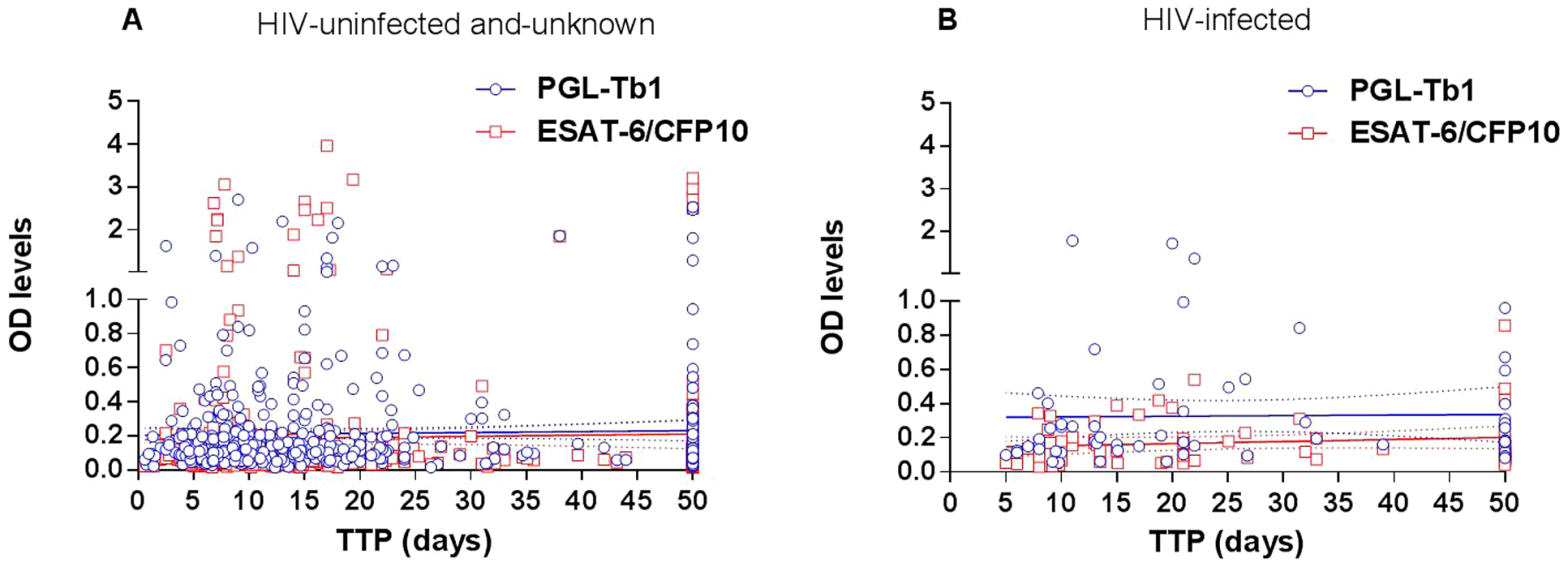
Impact of mycobacterial load and antibody responses stratified by HIV status. Relationship between individual time to positivity (TTP in days) of a liquid culture and individual serological tests based on optical density values against PGL-Tb1 antigen (blue spot) or ESAT-6/CFP10 (red spot) antigen in (A) the whole group of 270 HIV-uninfected and 293 HIV-unknown patients and in (B) the group of 68 HIV-infected patients with active pulmonary TB. The continuous lines represent the linear regression curve for anti-PGL-Tb1 antibody or for anti-ESAT-6/CFP10 antibody tested individuals; the hatched lines represent the 95% confidence intervals. **Footnotes**: HIV: Human Immunodeficiency Virus; OD: optical density; TTP: time to positivity.

This increased capacity to detect ATB patients might be also coupled with the absence of relationship between the antibody production and yield of mycobacteria in the patients' specimens. The yield of mycobacteria in the patients' specimens was measured as SM grade and the Time to Positivity (TTP) of the liquid culture [46]. As shown in **Figure 6A**, there was no correlation between the TTP (the number of days has been shown to be inversely related to the mycobacterial yield in the specimens) and the individual serum anti-PGL-Tb1 antibody level (R^2^ = 0.0007, *p* = 0.5449), or the anti-ESAT-6/CFP10 antibody level (R2 = 0.0005, *p* = 0.6013) among the groups of HIV-uninfected and HIV-unknown pulmonary TB patients. This absence of correlation was further demonstrated at each center and was independent of TB localization (data not shown). Similarly, the same absence of relationship (**Figure 6B**) was observed among the HIV-infected ATB patients: PGL-Tb1 (R^2^ = 0.0002, *p* = 0.9018), ESAT-6/CFP10 (R^2^ = 0.0189, *p* = 0.2786). Accordingly, we observed an absence of impact of the SM grade on the sensitivity of the serology tests in pulmonary TB patients, independent of the HIV status, as shown in **Table 12**.

**Table 12:** Sensitivity of the serology test based on the ELISA using the PGL-Tb1 and ESAT-6/CFP10 antigens and their cumulative results in active pulmonary TB patients stratified by SM grade and HIV status.

HIV status	n/N	SM (-)	n/N	SM scanty	n/N	SM (+)	n/N	SM (++)	n/N	SM (+++)
		% (95% CI)		% (95% CI)		% (95% CI)		% (95% CI)		% (95% CI)
**HIV-infected**										
**PGL-Tb1**	31/40	**77.5** (61.6–89.2)	4/4	**100** (39.8–100)	5/7	**71.4** (29.0–96.3)	6/10	**60.0** (26.2–87.8)	4/7	**57.1** (18.4–90.1)
*p SM (-) versus others*				*0.56*		*0.66*		*0.42*		*0.35*
**ESAT6/CFP10**	28/40	**70.0** (53.5–83.4)	2/4	**50.0** (68.0–93.2)	3/7	**42.9** (10.0–81.6)	6/10	**60.0** (26.2–87.8)	3/7	**42.9** (10. –81.6)
*p SM (-) versus others*				*0.58*		*0.21*		*0.71*		*0.21*
**Both antigens**	33/40	**82.5** (67.2–92.7)	4/4	**100** (39.8–100)	5/7	**71.4** (29.0–96.3)	6/10	**60.0** (26.2–87.8)	4/7	**57.1** (18.4–90.1)
*p SM (-) versus others*				*1.00*		*0.61*		*0.20*		*0.16*
**HIV-uninfected**										
**PGL-Tb1**	19/64	**29.7** (18.9–42.4)	9/24	**37.5** (18.8–59.4)	20/53	**37.7** (24.8–52.1)	20/50	**40.0** (26.4–54.8)	36/79	**45.6** (34.3–57.2)
*p SM (-) versus others*				*0.60*		*0.43*		*0.32*		*0.06*
**ESAT6/CFP10**	21/64	**32.8** (21.6–45.7)	6/24	**25.0** (9.8–46.7)	18/53	**34.0** (21.5–48.3)	13/50	**26.0** (14.6–40.3)	25/79	**31.7** (21.6–43.1)
*p SM (-) versus others*				*0.61*		*1.00*		*0.54*		*1.00*
**Both antigens**	28/64	**43.8** (31.4–56.7)	12/24	**50.0** (29.1–70.9)	26/53	**49.1** (35.1–63.2)	23/50	**46.0** (31.8–60.7)	43/79	**54.4** (42.8–65.7)
*p SM (-) versus others*				*0.64*		*0.58*		*0.85*		*0.24*
**HIV-unknown**										
**PGL-Tb1**	49/113	**43.4** (34.1–53.0)	9/14	**64.3** (35.1–87.2)	30/52	**57.7** (43.2–71.3)	11/39	**28.2** (15.0–44.9)	37/75	**49.3** (37.6–61.1)
*p SM (-) versus others*				*0.16*		*0.10*		*0.13*		*0.46*
**ESAT6/CFP10**	30/113	**26.6** (18.7–35.7)	6/14	**42.9** (17.7–71.1)	18/52	**34.6** (22.0–49.1)	12/39	**30.8** (17.0–47.6)	23/75	**30.7** (20.5–42.4)
*p SM (-) versus others*				*0.22*		*0.36*		*0.68*		*0.62*
**Both antigens**	56/113	**49.6** (40.0–59.1)	10/14	**71.4** (41.9–91.6)	36/52	**69.2** (54.9–81.3)	20/39	**51.3** (34.8–67.8)	45/75	**60.0** (48.4–71.2)
*p SM (-) versus others*				*0.16*		*0.0196*		*1.00*		*0.18*

**Footnotes**: TB: tuberculosis; HIV: human immunodeficiency virus; n/N = number of positive tests/number of tested sera in each group; SM: smear microscopy; Cut-off value for PGL-Tb1: OD = 0.130; Cut-off value for ESAT-6/CFP10: OD = 0.100; CI: confidence interval.

**Figure 7:**
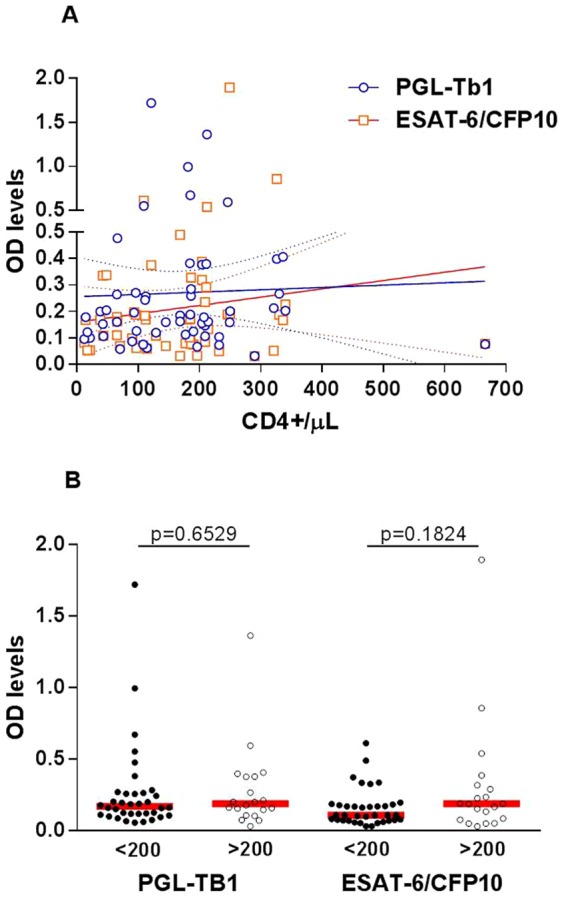
Impact of CD4 counts on antibody responses in HIV-infected active tuberculosis patients A. Correlation between individual CD4 cell counts and individual serological tests based on optical density values of ELISA against PGL-Tb1 antigen (blue spot) (R^2^ = 0.00103; p = 0.8141) or ESAT-6/CFP10 antigen (red spot) (R^2^ = 0.01574; p = 0.3569) in 53 HIV-infected ATB patients. The continuous lines represent the linear regression curve for anti-PGL-Tb1 antibody or for anti-ESAT-6/CFP10 antibody tested individuals; the hatched lines represent the 95% confidence intervals. B. Anti-PGL-Tb1 and anti-ESAT-6/CFP10 antibody levels stratified by CD4 cell counts among the 56 HIV-infected ATB patients. Horizontal red lines indicate the median value. **Footnotes**: OD: optical density.

Among the 56 HIV-infected ATB patients with reported concomitant CD4 cell counts [median (IQR): 179.0 cells/µl (93.75-211.8)], it was shown that the individual antibody level against both the anti-PGL-Tb1 and anti-EST-6/CFP10 antigen was not influenced by the degree of immunosuppression (**Figure 7A**). There was no correlation between the individual CD4 cell counts with the anti-PGL-Tb1 (R^2^ = 0.00103; *p* = 0.8141), or anti-ESAT-6/CFP10 (R^2^ = 0.01574; *p* = 0.3569) antibody levels. As shown in **Figure 7B**, similar anti-PGL-Tb1 antibody levels were found in HIV-infected ATB patients with CD4 cell counts higher or lower than 200/µl (OD = 0.171; 0.115-0.263 versus 0.190; 0.117-379, respectively; p = 0.6529). However, a lower anti-ESAT-6/CFP10 antibody level was found in HIV-infected ATB patients with CD4 cell counts less than 200/µl (OD = 0.110; 0.073-0.183) compared to those with CD4 cell counts higher than 200/µl (OD = 0.189; 0.080–0.312), but the difference was not statistically significant (p = 0.1824). Accordingly, the serology sensitivity among the HIV-infected ATB was not significantly different when patients were stratified by their CD4 cell counts using a threshold at 200/µl (**Table 13**).

**Table 13:** Comparison of the sensitivity of the TST and the serology test based on ELISA using each antigen and their cumulative results in HIV-infected active TB patients stratified by CD4 cell counts/µL (lower or above 200/µL).

	Percentage (95% CI)				
	n/N	<200 CD4 cell counts/µL	n/N	>200 CD4 cell counts/µL	*P*
		% (95% CI)		% (95% CI)	
**ELISA**					
**PGL-Tb1**	22/36	61.1 (43.5–76.9)	15/20	75.0 (50.9–91.3)	*0.3824*
**ESAT-6/CFP10**	22/36	61.1 (43.5–76.9)	14/20	70.0 (45.7–88.1)	*0.5713*
**Both antigens**	24/36	66.7 (49.0–81.4)	15/20	75.0 (50.9–91.3)	*0.5613*
**TST**	9/11	81.8 (48.2–97.7)	3/4	75.0 (19.4–99.4)	*1.000*

**Footnotes**: TB: tuberculosis; HIV: human immunodeficiency virus; n/N = number of positive tests/number of tested sera in each group; TST: tuberculin skin test (cut-off 5 mm); Cut-off value for PGL-Tb1: OD = 0.130; Cut-off value for ESAT-6/CFP10: OD = 0.100; CI: confidence interval.

### Comparison of the sensitivity of TST and ELISA for diagnosis of active TB

TST is the single diagnostic reference test for LTBI and cannot be used as a diagnostic test for ATB although it usually performed as a diagnostic adjunct tool for SM-negative patients with a high suspicion of ATB. We have compared the quantitative results of the two tests among the 277 ATB patients with concomitant TST and serology results.

**Figure 8:**
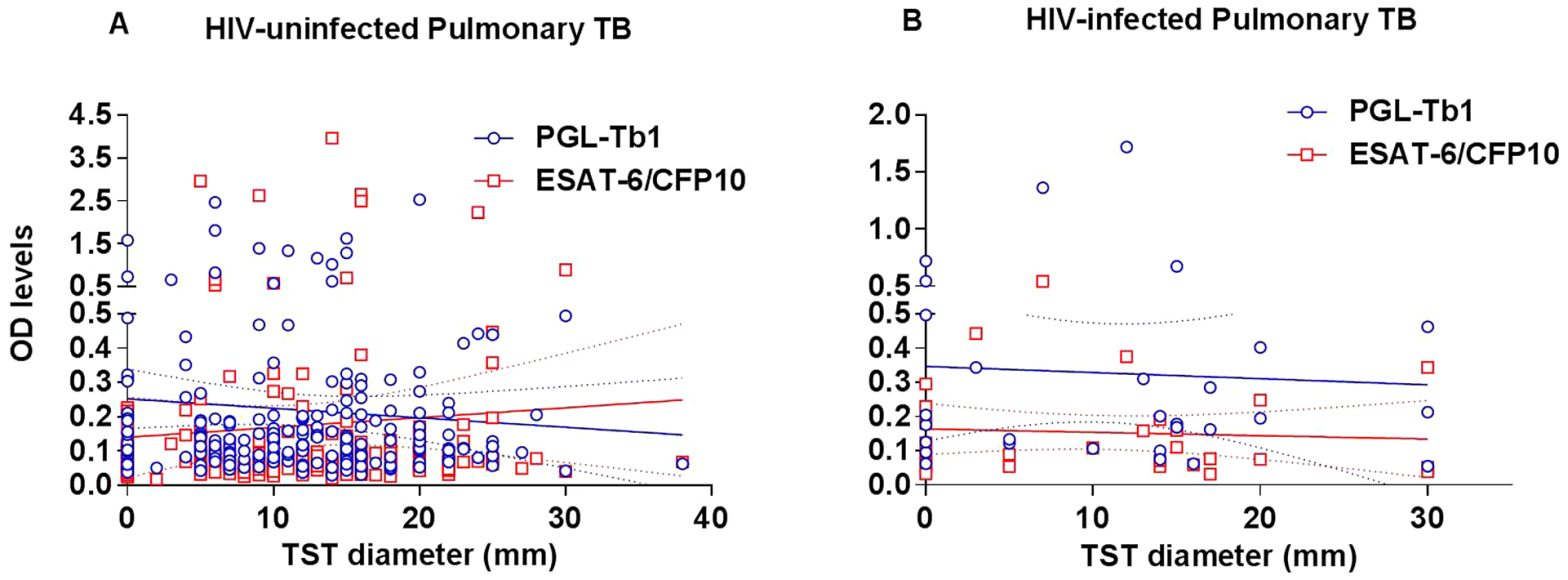
Correlation between the tuberculin skin test and antibody responses in active tuberculosis patients stratified by HIV status. Correlation between the individual tuberculin skin test diameter (in mm) and the individual serological tests based on optical density values of the ELISA against PGL-Tb1 antigen (blue spot) and ESAT-6/CFP10 (red spot) antigen in (A) the whole group of 127 HIV-uninfected and 119 HIV-unknown patients and in (B) the group of 30 HIV-infected patients with active pulmonary TB. The continuous lines represent the linear regression curve for anti-PGL-Tb1 antibody or for anti-ESAT-6/CFP10 antibody tested individuals; the hatched lines represent the 95% confidence intervals. **Footnotes**: HIV: Human Immunodeficiency Virus; TB: tuberculosis; TST: tuberculin skin test; OD: optical density.

As shown in **Figure 8A**, there was no significant correlation between the individual TST diameter and anti-PGL-Tb1 (R^2^ = 0.003504, p = 0.3800), or anti-ESAT-/CFP10 antibody levels (R^2^ = 0.002088, p = 0.4972) among the HIV-uninfected and HIV-unknown ATB patients. Similarly, there was also no significant correlation between the individual TST and PGL-Tb1 (R^2^ = 0.002048, p = 0.8123), or anti-ESAT-/CFP10 antibody levels (R^2^ = 0.005193, p = 0.7051) among the HIV-infected ATB patients (**Figure 8B**).

### Diagnostic values according to the clinical suspicion of TB (CSTB)

It was also interesting to evaluate the association between the two serological tests based on ELISA results and the CSTB described in ATB patients stratified by HIV status and TB localization; however, no impact of the CSTB on the serology results was observed in pulmonary or extrapulmonary ATB, independent of their HIV status (data not shown).

## Discussion

We are presenting the results of the serology study using an ELISA format based on a phenolic glycolipid (PGL-Tb1) or fusion protein (ESAT6/CFP10). This study was the third part of a prospective multi-centric trial designed to investigate the performance of a diagnostic toolbox for ATB in India, a highly TB-endemic country. The two previous reports involved different diagnostic tools such as the clinical and radiological findings, microbiological results [46], and *in vitro* cell-mediated immune responses as measured by the QuantiFERON-TB Gold in Tube and TST [47]. Using the same populations of ATB patients and non-ATB individuals, our aim was to assess the accuracy of two serological tests *per se* and as additional tools to support ATB diagnosis.

Although no current guideline recommends the use of serological assays for detecting ATB, dozens of commercial kits are sold, mostly in developing countries [63]. Scientific evidence of published data on commercial serological tests for both pulmonary and extrapulmonary TB diagnosis reported inconsistent estimates of sensitivity and specificity [43], [44]. Moreover, poor overall quality of these tests has been attributed to an unsatisfactory patient selection methodology [64]. Based on these findings, cost-effective data and expert opinion, the World Health Organization (WHO) has issued a recommendation against the use of currently available serological tests for TB diagnosis, but has stressed the importance of continued research on these and other tests that could provide quick and accurate TB diagnosis [65].

Insufficient serological results are available for ATB sputum smear-negative patients with HIV co-infection [45], [66]–[73]. Moreover, these results are based on the use of protein antigens that may affect the accuracy of the assays. The approach of using non-protein antigens (phosphoglycolipid, lipo-oligosaccharide, diacyl-trehalose or phenolic glycolipid) may overcome this drawback [32]. In fact, in HIV–TB co-infected patients from low TB endemic countries, the sensitivity of a serological test based on an ELISA using a glycolipid was similar [14] or even higher compared to an assay based on protein antigens [39], [74]. Moreover, these mycobacterial-derived products seem to be species specific, without any influence from previous BCG vaccination [75] or co-existing mycobacteriosis due to non-tuberculosis mycobacteria infection [13].

In our current multi-centric study performed in India, we confirmed the data generated in non-endemic TB countries showing a high potential of serological tests with one glycolipid antigen (PGL-Tb1) in HIV-TB co-infected patients [39]. In addition, our present data showed that in ATB patients, the IgG antibody levels to one single antigen (PGL-Tb1 or ESAT-6/CFP10) were independent of the SM status and grade and were significantly higher than the levels observed in all healthy controls at relatively low and high risk of LTBI, except the cured TB patients. Nonetheless, the IgG antibody levels against both the PGL-Tb1 and ESAT-6/CFP10 antigen in the healthy controls varied according to the site of recruitment and the extent of *M. tuberculosis* exposure. As reported previously, the varying degree of TB infectiousness was demonstrated among the ATB patients enrolled at the different sites of recruitment [46], and consequently, a higher prevalence of LTBI, as measured with the TST and QFT-GIT responses, was observed in the close contacts recruited at the sites where the ATB patients were more infectious [47]. This indicates that the ATB incidence in a given country and region has a marked influence on the cell-mediated responses and antibody responses to *M.tuberculosis* specific antigens reflecting repeated exposure to the pathogen [37].

Among the groups of ATB patients, there was no significant difference of antibody levels or test sensitivity between the HIV-uninfected and HIV-unknown ATB patients. This is most likely due to the fact that these two populations of ATB patients were similar regarding their HIV status, as previously reported [46], [47].

In contrast, the anti-PGL-Tb1 and anti-ESAT6/CFP10 antibody levels were significantly higher in HIV-infected sera than in HIV-uninfected (p<0.0001), or in HIV-unknown (p<0.0001) sera from ATB patients. Thus, although the majority of HIV-infected ATB patients in our study had advanced immunosuppression, it was shown that the anti-PGL-Tb1 antibody production was not dependent on the degree of immunosuppression. On the other hand, there was a trend for the anti-ESAT-6/CFP10 fusion protein antibody response to be more dependent on the degree of immunosuppression (higher antibody levels were found in HIV-TB co-infected patients with more than 200/µl CD4 cell counts compared to those with lower counts). However, the difference was not significant, probably due to the low number of patients with available CD4 counts.

As recently demonstrated, the absence of effect upon the anti-PGL-Tb1 response could be linked to the glycolipid structure of the PGL-Tb1 antigen and its presentation via the CD1 pathway for lymphocyte activation [33], [34]. Indirect evidence that the canonical presentation pathway involving CD4T-lymphocytes is not involved in the anti-glycolipid antibody response is illustrated by the absence of a direct correlation between the CD4 T-lymphocyte counts and the individual IgG antibody production, as shown in our study. Likewise, evidence has been published demonstrating that a subpopulation of CD4 T-lymphocytes involved in the CD1-restricted glycolipid antigen presentation pathway in leprosy [76] and TB patients [77], [78] persists in patients with AIDS [79]. T-lymphocyte independence of the antibody response to another specific mycobacterial phenolic glycolipid antigen (the PGL-1 antigen being specific for *M.leprae*) has been shown in lepromatous leprosy patients, also when very high anti-PGL-1 antibody levels (both IgM and IgG) were found in the absence of a specific T-cell mediated immune response [80]. It was also shown there was no relationship between TST results and anti-PGL-Tb1 antibody levels in ATB patients, similar to the results shown in the present study (**Fig.8**). In the lepromatous leprosy patients, a good correlation between IgM and IgG anti-PGL-1 antibody levels and bacteriological yield (as expressed by the bacteriological index performed in skin biopsies) has been also described [81]. However in the context of pulmonary TB, no significant association between the proportion of anti-PGL-Tb1 positive results and bacillary load was found. Our results confirmed the absence of a relationship between the bacillary load and antibody levels: no significant correlation was observed between the Time to Positivity (in days) of a liquid culture and the anti-PGL-Tb1 or anti-ESAT-6/CFP10 antibody levels in the same ATB patients, independent of the HIV status. Similarly, the sensitivity of the serological test based on an ELISA using each antigen was not influenced by the SM status or grade evaluated among the specimen samples of the pulmonary ATB patients.

With an overall specificity of around 90%, the sensitivity of the two serological tests, using either the PGL-Tb1 or the ESAT-6/CFP10 antigen, was statistically higher in the HIV-TB co-infected patients compared to the HIV-uninfected or HIV-unknown TB patients, independent of the disease localization [39]. The overall sensitivity for ATB using the cumulative results obtained with the two antigens was always higher than using one single antigen, but with a statistical significant reduction of specificity, confirming previous studies [14], [45], [61]. Nevertheless, the sensitivity of the serological tests for ATB in HIV-infected subjects was 75.5% with a significant negative LR (94.9%). Lower sensitivity was shown among the HIV-uninfected (50.9%) and HIV-unknown ATB patients (54.1%).

The sensitivity of the SM for diagnosing ATB was variable and dependent on the HIV status and localization of the disease, being very low among the whole group of extrapulmonary ATB (17%) and among the whole group of HIV-infected ATB patients (31.4%). In the present study, the serology using either the glycolipid or the fusion protein antigen was able to detect an equal number of ATB patients, independent of SM score and HIV status. Thus, the combination of SM-positive results with the serological tests based on single or multiple antigens (cumulative results) improved the case findings in ATB patients. This higher sensitivity could be related to the independence of antibody production of disease localization, mycobacterial load of the ATB patient's specimens and the degree of immunosuppression.

The varying degree of the physicians' clinical suspicion of TB did not influence the accuracy of the two serological tests and other immunological tools [47], in contrast to the clinical symptoms, radiological findings and microbiological tools [46]. These results might be associated with the absence of impact of duration and severity of TB disease on the B-cell immune responses in ATB patients.

In conclusion, from a clinical viewpoint, our study showed that the combined use of the SM test and the two serological tests led to an increase of sensitivity for ATB diagnosis in those with extrapulmonary localization and HIV co-infection. Therefore, serological assays using the cumulative results obtained with both a phenolic glycolipid antigen such as the PGL-Tb1 and a fusion protein such as the ESAT-6/CFP10 may be evaluated as a potentially interesting additional tool for diagnosing ATB in HIV-infected patients in high TB-endemic countries.
